# HLA-G expression in non-small cell lung cancer: prognostic significance and interplay with PD-L1 and CD8^+^ tumor-infiltrating lymphocytes

**DOI:** 10.3389/fimmu.2026.1732852

**Published:** 2026-06-12

**Authors:** Giulia Querzoli, Giuseppe Bogina, Marcella Marconi, Nicola Tumino, Paola Vacca, Luisella Righi, Sara Pilotto, Anna Caliò, Luca Cima, Stefano Gobbo, Matthew J. Cecchini, Gaetano Paolino, Francesco Ciompi, Roberto S. Accolla, Aldo Scarpa, Gianluigi Lunardi, Emanuela Marcenaro, Lorenzo Moretta, Giuseppe Zamboni, Enrico Munari

**Affiliations:** 1 Pathology Unit, IRCCS Azienda Ospedaliero-Universitaria di Bologna, Bologna, Italy; 2 Pathology Unit, IRCCS Sacro Cuore Don Calabria Hospital, Negrar di Valpolicella, VR, Italy; 3 Innate Lymphoid Cells Unit, Immunology Research Area, Bambino Gesù Children’s Hospital, IRCCS, Rome, Italy; 4 Pathology Unit, Department of Oncology, University of Torino at San Luigi Hospital, Orbassano, Italy; 5 Department of Engineering for Innovation Medicine (DIMI), University of Verona and Verona University Hospital Trust, Verona, Italy; 6 Department of Diagnostic and Public Health, Section of Pathology, University of Verona and Verona University Hospital Trust, Verona, Italy; 7 Pathology Unit, Verona University Hospital Trust, Verona, Italy; 8 Department of Pathology and Laboratory Medicine, Schulich School of Medicine and Dentistry, Western University, London, ON, Canada; 9 Pathology Unit, ASST Spedali Civili di Brescia, Brescia, Italy; 10 Department of Pathology, Radboud University Medical Center, Nijmegen, Netherlands; 11 Laboratories of General Pathology and Immunology “Giovanna Tosi”, Department of Medicine and Technological Innovation, University of Insubria, Varese, Italy; 12 Laboratory Medicine, IRCCS Sacro Cuore Don Calabria Hospital, Negrar di Valpolicella, VR, Italy; 13 Department of Experimental Medicine, University of Genova, Genova, Italy; 14 IRCCS Azienda Ospedaliera Metropolitana (AOM), Genova, Italy; 15 Tumor Immunology Unit, Bambino Gesù Children’s Hospital, IRCCS, Rome, Italy

**Keywords:** HLA-G, immune evasion, non-small cell lung cancer, PD-L1, tumor-infiltrating lymphocytes

## Abstract

**Introduction:**

HLA-G is a non-classical major histocompatibility complex class I molecule with potent immunosuppressive activity and is increasingly recognized as an immune-checkpoint axis in cancer. Its prognostic significance in non-small cell lung cancer (NSCLC), particularly in relation to PD-L1 expression and CD8^+^ tumor-infiltrating lymphocytes (TILs), remains incompletely defined.

**Methods:**

We retrospectively analyzed 314 surgically resected NSCLCs assembled in tissue microarrays and stained for HLA-G, PD-L1, and CD8. HLA-G and PD-L1 were scored as positive when ≥1% of tumor cells showed membranous staining, whereas CD8^+^ TIL density was digitally quantified and dichotomized using the cohort median (≥575 cells/mm²). Associations with clinicopathological variables and outcomes were assessed by Kaplan–Meier analysis and multivariable Cox regression.

**Results:**

HLA-G was expressed in 50 of 314 tumors (16%), PD-L1 in 106 of 314 (33.8%), and high CD8 density in 160 of 314 (51%). In HLA-G-negative tumors, high CD8^+^ TIL density was associated with significantly prolonged disease-free survival (DFS) and overall survival (OS). In the overall cohort, the combined HLA-G-negative/CD8-high phenotype retained independent favorable prognostic significance for both DFS and OS. By contrast, CD8 density did not significantly stratify outcome in HLA-G-positive tumors, although these subgroup analyses were limited by small sample size.

**Discussion:**

In combined biomarker analyses, the favorable prognostic effect of CD8^+^ TILs in PD-L1-negative tumors was maintained only when HLA-G was also absent. Within the PD-L1-positive/HLA-G-negative subgroup, high CD8 density was independently associated with improved DFS but not OS. Integrating HLA-G with PD-L1 and CD8 assessment may refine prognostic stratification and help identify patients who could benefit from HLA-G-targeted strategies, alone or in combination with PD-1/PD-L1 blockade.

## Introduction

Human leukocyte antigen G (HLA-G) is a non-classical major histocompatibility complex (MHC) class I molecule belonging to the HLA-class Ib family. Unlike the highly polymorphic HLA-class Ia molecules that are broadly expressed on most nucleated cells, HLA-G and other class Ib molecules (HLA-E, HLA-F, and HLA-H) have limited polymorphism and a restricted tissue distribution (1).

Physiological HLA-G expression is mainly confined to fetal tissues such as cytotrophoblasts, where its main function is to induce immune tolerance to protect the fetus during pregnancy, and it is barely detectable in adult tissues aside from thymic medulla, cornea, pancreas, and erythroid and endothelial precursors (2).

Seven HLA-G isoforms are generated by alternative splicing of the primary HLA-G mRNA: four membrane-bound isoforms (HLA-G1–G4) and three soluble isoforms (HLA-G5–G7) (3). In addition, membrane-bound HLA-G1 can be shed by metalloproteinases to produce a soluble “shed” form (4).

HLA-G exerts potent immunosuppressive effects by binding inhibitory receptors such as ILT2, ILT4, and KIR2DL4 expressed on natural killer (NK) cells, CD8^+^ cytotoxic T lymphocytes, and antigen-presenting cells (5, 6).

HLA-G expression, while absent or very low in most normal adult tissues, is frequently up-regulated in malignant cells. It was first observed in melanoma and subsequently in a broad range of solid and hematological malignancies, including breast, kidney, ovary, lung, and colorectal cancers (1, 7–10). Increased HLA-G expression is often associated with higher tumor stage, lymph-node metastasis, and poor survival; a recent meta-analysis of 25 studies (4871 patients) reported that positive HLA-G immunohistochemistry was associated with significantly worse overall survival across solid tumors, particularly gastric, pancreatic, and colorectal cancers (11).

As previously mentioned, increasing evidence shows that HLA-G can effectively suppress the cytotoxic activity of immune cells such as T cells and NK cells, highlighting its potential role as a new immune checkpoint. Nevertheless, the differences and relationships between HLA-G and established immune checkpoint molecules such as PD-1/PD-L1 are still not well understood (12).

In our previous work (13), we demonstrated that in resected NSCLC the median density of CD8^+^ tumor-infiltrating lymphocytes is significantly associated with both disease-free and overall survival in PD-L1^-^ tumors, but not in PD-L1^+^ tumors. Building on these findings, in this work we sought to determine whether HLA-G shows a similar modulatory effect.

## Materials and methods

### Patients characteristics

The study cohort included a consecutive series of patients who had undergone surgical resection for primary NSCLC at the IRCCS Sacro Cuore Don Calabria Hospital of Negrar, Verona, Italy, between 2003 and 2018, and for whom slides and paraffin-embedded tissue blocks were available. None of the patients received neoadjuvant chemotherapy or radiotherapy before thoracic surgery. Tumors were classified according to the 2020 WHO classification, and staging was based on the 8th edition of the AJCC TNM staging manual (14).

The study was approved by the Ethical Committee (3130_OPBG_2023 and 25046, 4/26/2021 Clinical Research of Verona and Rovigo) and conducted according to the tenants of the declaration of Helsinki.

### Tissue samples and immunohistochemistry

For each case, all hematoxylin and eosin–stained slides were reviewed for confirmation of diagnosis; one paraffin-embedded block was then selected for tissue micro-array (TMA) preparation. Five cores with a diameter of 1 mm were obtained from different areas of the tumor in each block and randomly numbered from 1 to 5. Consecutive, 5-μm sections were then cut from each block and stained for CD8 (clone SP57, Ventana Medical Systems, Tucson, AZ), PD-L1 (clone SP263, Ventana Medical Systems, Tucson, AZ), HLA-G (clone 4H84, Santa Cruz Biotechnology, Dallas, Texas) on an automated staining platform (Benchmark Ultra [Ventana Medical Systems]). An OptiView DAB IHC Detection Kit (Ventana Medical Systems) and an OptiView Amplification Kit (Ventana Medical Systems) were used according to the manufacturer’s recommendations for visualization of the primary anti CD8, anti PD-L1 and anti HLA-G antibody. Stained sections were scanned with a Ventana iScan HT slide scanner (Ventana Medical Systems). PD-L1 and HLA-G expression was evaluated independently by two pathologists (EM and GQ) and calculated as the percentage of membrane staining on tumor cells with any intensity. In case of disagreement, a third pathologist reviewed the case and a consensus score was assigned for final analysis; the final score was calculated as the average of all available cores. Cases were considered positive for PD-L1 and/or HLA-G if at least 1% of the tumor cells showed membranous immunoexpression. CD8^+^ TILs within tumor nests were automatically counted using QuPath version 0.2.0 (15).

### Statistical analysis

Data importation and statistical analysis were performed using STATA/IC software for Windows, version 14.0.

Chi-square tests were used to analyze the association between HLA-G and CD8-positive cells, clinicopathological variables, and PD-L1 in neoplastic cells.

Disease-free survival (DFS) was calculated from the date of primary surgical treatment to the date of recurrence or the date of death from any cause. Overall survival (OS) was calculated from the date of primary surgical treatment to the date of death from any cause or the date of the last follow-up observation. For a patients with stage IV disease, only OS was considered. On the last follow-up visit, patients alive with no sign of relapse were censored for DFS, while patients alive regardless of relapse were censored for OS. Long-term survivors were censored at 120 months of follow-up. Cumulative incidence of DFS and OS in the groups was described by the Kaplan–Meier method and compared with the use of the log-rank test.

The Cox proportional hazard regression model was used to evaluate the associations between clinicopathological factors and clinical outcome, with a two-sided *P* value <0.05 considered as statistically significant.

Interobserver agreement for PD-L1 and HLA-G scoring was assessed using Cohen’s kappa statistic.

## Results

### Patient characteristics

A total of 314 patients with histologically confirmed resected NSCLC were included in the study. The median age at diagnosis was 70 years (range: 40–86), and the majority were male (221 patients, 70.4%). Adenocarcinoma was the predominant histologic subtype, accounting for 71.4% of cases. Most patients underwent lobectomy (77.1%) and were diagnosed with stage I or II disease (76.1%). Adjuvant chemo-radiation therapy had been administered to 44 patients. Patient characteristics are summarized in **Table 1**.

**Table 1:** Clinicopathological characteristics of patients with NSCLC, stratified according to HLA-G expression.

Variables	Overall (%)	HLA-G		P value
		< 1% (%)	≥ 1% (%)	
Patients	314	264 (84)	50 (16)	
Sex				
Male	221 (70.4)	186 (84.2)	35 (15.8)	*0.95*
Female	93 (29.6)	78 (83.9)	15 (16.1)	
Age (years)				
≤ 70	163 (51.9)	132 (81)	31 (19)	*0.12*
> 70	151 (48.1)	132 (87.4)	19 (12.6)	
Histology				
Adenocarcinoma	224 (71.4)	188 (83.9)	36 (16.1)	*0.68*
SCC	72 (22.9)	62 (86.1)	10 (13.9)	
Others	18 (5.7)	14 (77.8)	4 (22.2)	
Surgery				
Wedge/Segmentectomy	56 (17.8)	46 (82.1)	10 (17.9)	*0.61*
Lobectomy	244 (77.7)	205 (84)	39 (16)	
Pneumonectomy	14 (4.5)	13 (92.9)	1 (7.1)	
TNM Stage				
I	159 (50.6)	128 (80.5)	31 (19.5)	*0.20*
II	80 (25.5)	72 (90)	8 (10)	
III	53 (16.9)	47 (88.7)	6 (11.3)	
IV	16 (5.1)	13 (81.2)	3 (18.8)	
Unknow	6 (1.9)	4 (66.7)	2 (33.3)	
Adjuvant Treatment				
No	242 (77.1)	201 (83.1)	41 (16.9)	*0.41*
Yes	44 (14)	40 (90.9)	4 (9.1)	
Unknow	28 (8.9)	23 (82.1)	5 (17.9)	
PD-L1 (1% threshold)				
Negative	208 (66.2)	180 (86.5)	28 (13.5)	*0.09*
Positive	106 (33.8)	84 (79.2)	22 (20.8)	
CD8 (median threshold)				
Negative	154 (49)	135 (87.7)	19 (12.3)	*0.09*
Positive	160 (51)	129 (80.6)	31 (19.4)	

### HLA-G expression and clinicopathological correlates

HLA-G was expressed in 50 of 314 cases (16%) while PD-L1 was positive in 106 of 314 cases (33.8%). Although assessment of interobserver variability was not a primary aim of the study, we observed a good level of agreement among the pathologists, with Cohen’s κ values greater than 0.6.

The average and median counts of CD8^+^ cells were 750 (SD: 635) and 575 (range: 10–3015) cells per mm², respectively. The median CD8 count was used as the cut-off, and cases with ≥575 CD8^+^ cells per mm² were classified as positive. Clinicopathological characteristics stratified by HLA-G expression are summarized in **Table 1**. There was a trend toward a statistically significant association between HLA-G expression in tumor cells and both the density of CD8^+^ lymphocytes and PD-L1 expression (P = 0.09 for each).

**Figure 1:**
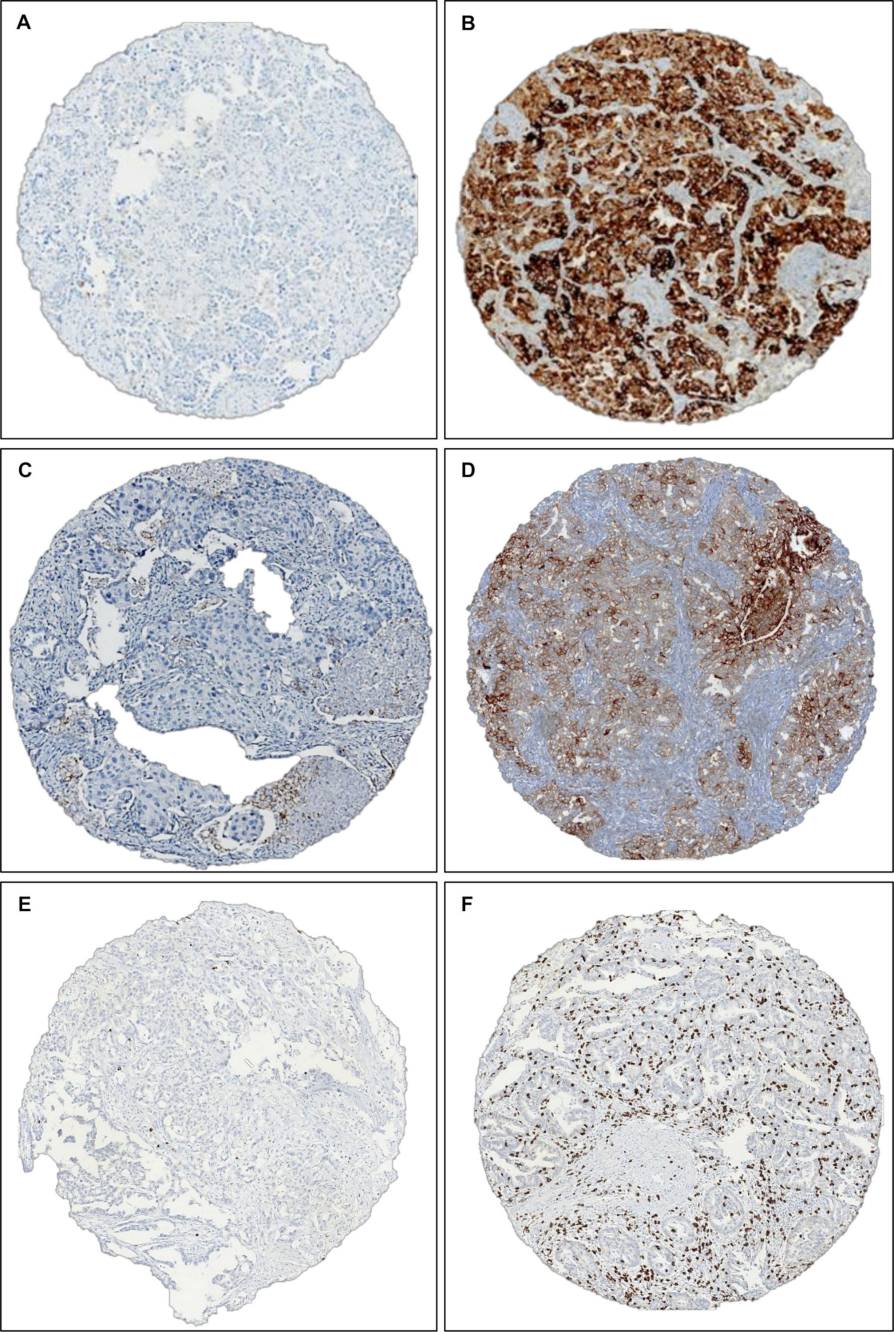
Representative examples of immunostaining for HLA-G [**(A)** negative, **(B)** positive], PD-L1 [**(C)** negative, **(D)** positive] and CD8 [**(E)** < 575 cells/mm^2^, **(F)** ≥ 575 cells/mm^2^].

**Figure 1** shows representative sections stained for HLA-G, PD-L1 and CD8.

Clinicopathological characteristics stratified by PD-L1 expression are summarized in **Supplementary Table 1**.

### Clinical outcomes

Disease progression data were available for 233 patients from the overall cohort. Sixty-five patients were lost to follow-up, and 16 were excluded due to the presence of distant metastases at diagnosis. Among the evaluable patients, 94 experienced disease progression. The median time to relapse was 12 months (95% CI: 10–16).

Overall survival analysis was feasible in 293 patients. At the time of analysis, 120 patients had died, with a median time to death of 27 months (95% CI: 23–30). Among patients who were alive and disease-free at the time of analysis, the median follow-up was 50 months (95% CI: 41–56).

Survival analysis for CD8, PD-L1 and HLA-G are reported in **Supplementary Figure 1**.

### Survival analysis based on CD8^+^ lymphocyte density and HLA-G expression in tumor cells

**Figure 2:**
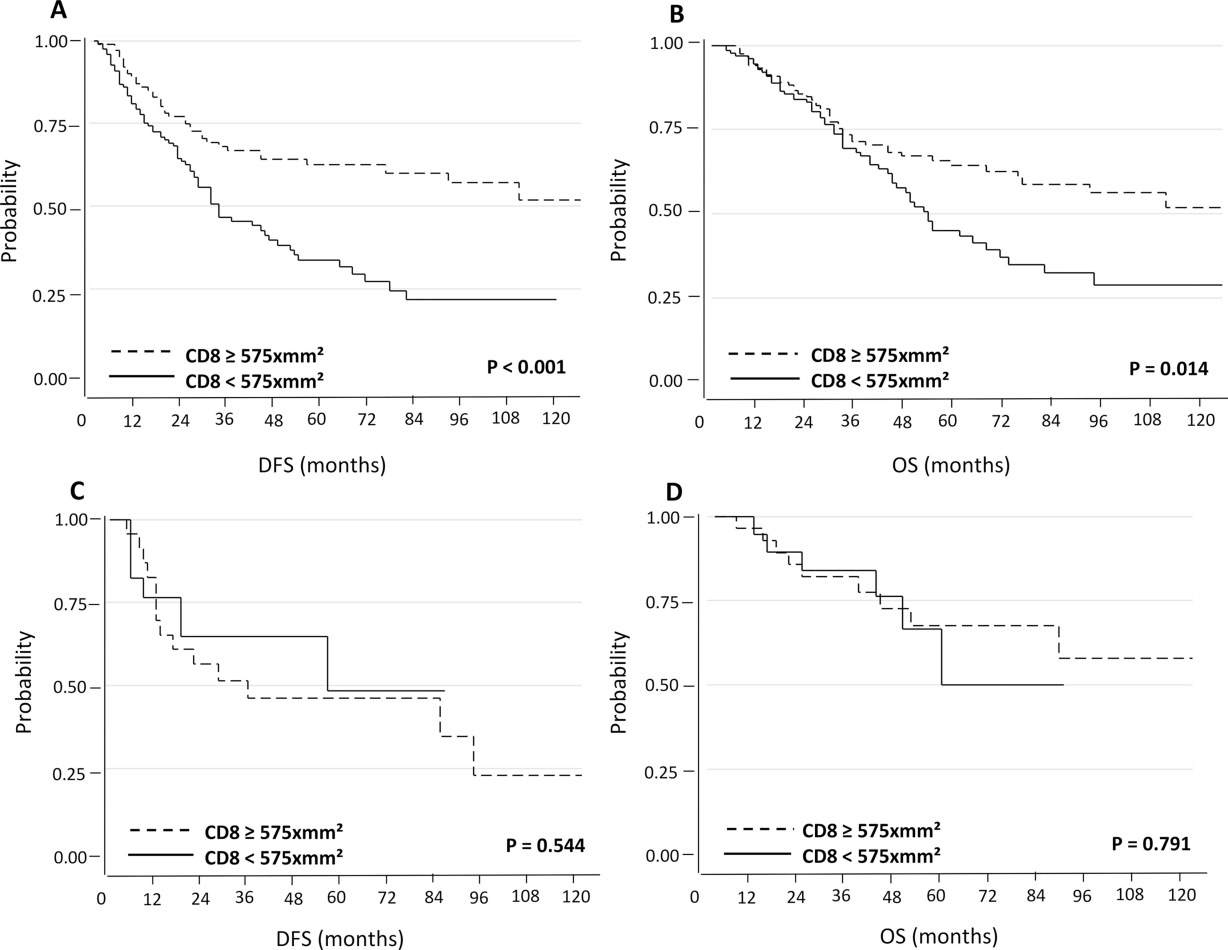
Kaplan–Meier curves for disease-free survival [DFS; **(A, C)**] and overall survival [OS; **(B, D)**] according to CD8^+^ T-cell density (≥575 vs <575 cells/mm²) in patients with HLA-G^-^ **(A, B)** and HLA-G^+^ tumors **(C, D)**.

In the subgroup of tumors negative for HLA-G, high CD8^+^ lymphocyte density was associated with significantly improved disease-free survival (DFS) and overall survival (OS) (**Figures 2A, B**). The HLA-G^-^/CD8high phenotype showed favorable prognostic impact in the overall cohort in multivariate analysis: HR = 0.35 (95% CI: 0.21-0.57) for DFS and 0.6 (95% CI: 0.39-0.92) for OS (**Table 2**).

**Table 2:** Multivariate Cox regression analysis for disease-free survival (DFS) and overall survival (OS) according to the combined HLA-G^−^/CD8-high phenotype in the overall cohort.

Variables	DFS			OS		
	HR	95% CI	P value	HR	95% CI	P value
Sex						
Male	1			1		
Female	0.74	0.46-1.19	*0.22*	0.68	0.43-1.09	*0.12*
Age (years)						
≤ 70	1			1		
> 70	0.66	0.40-1.07	*0.09*	1.05	0.66-1.67	*0.81*
Histology						
Adenocarcinoma	1			1		
Others	0.55	0.33-0.91	*0.02*	0.92	0.58-1.44	*0.72*
Surgery						
Wedge/Lobectomy	1			1		
Pneumonectomy	0.70	0.40-1.22	*0.21*	0.80	0.51-1.25	*0.33*
Adjuvant Treatment						
No	1			1		
Yes	0.77	0.43-1.37	*0.37*	1.15	0.64-2.06	*0.62*
TNM Stage						
II-IV	1			1		
I	0.14	0.08-0.26	*<0.001*	0.32	0.19-0.52	*<0.001*
HLA-G^-^/CD8high						
No	1			1		
Yes	0.35	0.21-0.57	*<0.001*	0.60	0.39-0.92	*0.021*

In contrast, no significant differences in DFS or OS were observed in the subgroup of patients with HLA-G^+^ tumors (**Figures 2C, D**). Of note, the HLA-G^+^ subgroups were relatively small, which limits the statistical power of these analyses, particularly for the negative survival comparisons within HLA-G^+^ tumors (**Supplementary Table 2**).

### Survival analysis according to CD8^+^ lymphocyte density, PD-L1 and HLA-G expression

**Figure 3:**
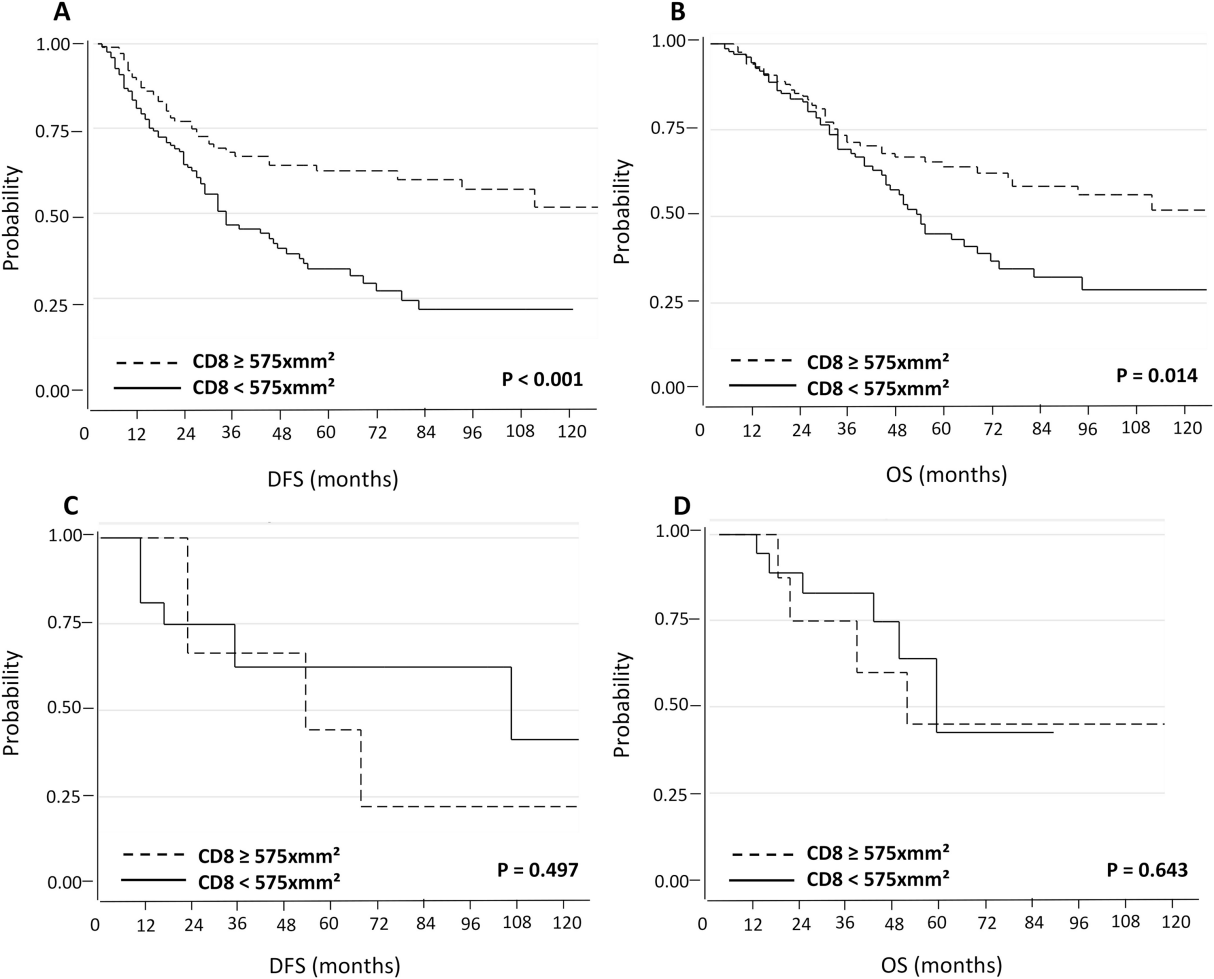
Kaplan–Meier curves for DFS **(A, C)** and OS **(B, D)** according to CD8^+^ T-cell density (≥575 vs <575 cells/mm²) in patients with tumors negative for both PD-L1 and HLA-G **(A, B)** and in patients with PD-L1^-^/HLA-G^+^ tumors **(C, D)**.

**Figure 4:**
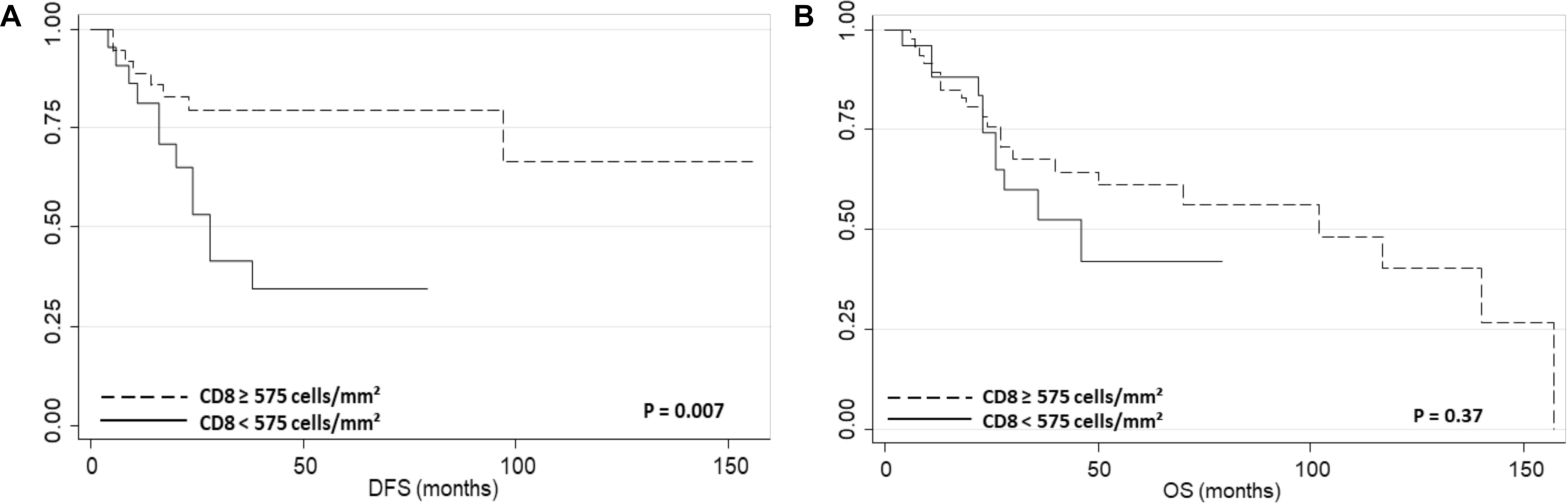
Kaplan-Meier curves for DFS **(A)** and OS **(B)** according to CD8^+^ T-cell density (≥575 vs <575 cells/mm²) in patients PD-L1^+^/HLA-G^-^ tumors.

In patients with PD-L1^–^/HLA-G^–^ tumors, cases with high CD8^+^ TILs density showed a significantly longer DFS and OS compared with cases with low CD8^+^ TILs density (**Figures 3A, B**). The favorable prognostic impact of CD8^+^ infiltration remained significant in multivariate analysis: HR = 0.37 (95% CI: 0.21-0.65) for DFS and 0.43 (95% CI: 0.24-0.76) for OS (**Table 3**). In contrast, among patients with PD-L1^-^/HLA-G^+^ tumors, high CD8^+^ density was not associated with improved DFS or OS (**Figures 3C, D**). Finally, within the PD-L1^+^/HLA-G^-^ subgroup, tumors with high CD8^+^ TILs density were associated with prolonged DFS compared with cases with low CD8^+^ TILs density (**Figure 4A**), but this benefit was not observed in OS (**Figure 4B**). **Table 4** shows multivariate Cox regression analysis for DFS and OS in patients with tumors negative for HLA-G and positive for PD-L1.

**Table 3:** Multivariate Cox regression analysis for DFS and OS in patients with tumors negative for both HLA-G and PD-L1.

Variables	DFS			OS		
	HR	95% CI	P value	HR	95% CI	P value
Sex						
Male	1			1		
Female	0.70	0.41-1.18	*0.18*	0.63	0.36-1.11	*0.11*
Age (years)						
≤ 70	1			1		
> 70	0.93	0.55-1.60	*0.81*	1.23	0.69-2.18	*0.70*
Histology						
Adenocarcinoma	1			1		
Others	0.88	0.49-1.61	*0.69*	0.75	0.39-1.44	*0.39*
Surgery						
Wedge/Lobectomy	1			1		
Pneumonectomy	0.35	0.08-1.47	*0.15*	0.57	0.13-2.46	*0.45*
Adjuvant Treatment						
No	1			1		
Yes	0.89	0.44-1.80	*0.75*	0.98	0.46-2.08	*0.87*
TNM Stage						
II - IV	1			1		
I	0.24	0.13-0.43	*<0.001*	0.32	0.17-0.59	*<0.001*
CD8						
< 575xmm²	1			1		
≥ 575xmm²	0.37	0.21-0.65	*<0.001*	0.43	0.24-0.76	*<0.001*

**Table 4:** Multivariate Cox regression analysis for DFS and OS in patients with tumors negative for HLA-G and positive for PD-L1.

Variables	DFS			OS		
	HR	95% CI	P value	HR	95% CI	P value
Sex						
Male	1			1		
Female	0.49	0.11-2.04	*0.32*	1.27	0.36-4.46	*0.70*
Age (years)						
≤ 70	1			1		
> 70	0.29	0.08-1.02	*0.05*	0.61	0.20-1.79	*0.37*
Histology						
Adenocarcinoma	1			1		
Others	0.93	0.34-2.54	*0.90*	1.07	0.46-2.46	*0.86*
Surgery						
Wedge/Lobectomy	1			1		
Pneumonectomy	0.85	0.30-2.41	*0.77*	0.81	0.36-1.82	*0.73*
Adjuvant Treatment						
No	1			1		
Yes	0.67	0.16-2.77	*0.58*	0.80	0.23-2.81	*0.73*
TNM Stage						
II - IV	1			1		
I	0.19	0.04-0.79	*0.022*	0.16	0.05-0.53	*0.003*
CD8						
< 575xmm²	1			1		
≥ 575xmm²	0.28	0.10-0.76	*0.013*	0.97	0.37-2.56	*0.96*

## Discussion

Accumulating evidence indicates that high densities of tumor-infiltrating lymphocytes (TILs), particularly CD8^+^ cytotoxic T-cells, are strongly associated with improved clinical outcomes in NSCLC. This underscores their prognostic and potential predictive value (16).

In this work, we found that CD8^+^ TIL density is associated with prolonged survival in patients with HLA-G^-^ tumors, but not in those with HLA-G^+^ tumors. We then examined the prognostic value of CD8^+^ TILs across combined biomarker subgroups and observed that CD8^+^ TILs confer a survival benefit in tumors negative for both PD-L1 and HLA-G, but not in PD-L1^-^ tumors that express HLA-G. Moreover, we show that in tumors that are PD-L1^+^ and HLA-G^-^, high CD8^+^ TILs density was associated with improved disease-free survival; however, this benefit did not translate into a significant improvement in overall survival.

These findings extend previous observations that HLA-G expression is associated with advanced disease stage and poorer outcomes, suggesting that HLA-G mediates immune escape by inhibiting cytotoxic T cell activity within the tumor microenvironment (11).

In PD-L1^-^ tumors, the fact that CD8^+^ infiltration retains prognostic value only when HLA-G is also absent is consistent with a model of compensatory immune suppression in which distinct checkpoint pathways can independently neutralize cytotoxic T-cell function. In a PD-L1^-^ setting, HLA-G expression can provide an alternative dominant inhibitory signal on cytotoxic lymphocytes and antigen-presenting cells, potentially reshaping the microenvironment toward tolerance. This would explain why PD-L1^−^/HLA-G^+^ tumors behave as a biologically distinct phenotype in which CD8^+^ cells are present but functionally restrained, whereas PD-L1^−^/HLA-G^−^ tumors represent a permissive context in which CD8^+^ infiltration translates into meaningful antitumor control and improved outcomes. Conceptually, these data suggest that effective immune surveillance in NSCLC may require the simultaneous absence of multiple non-overlapping inhibitory axes, because blockade or absence of one checkpoint (PD-L1) can be “bypassed” by another (HLA-G).

We also examined the reciprocal scenario (PD-L1^+^/HLA-G⁻ tumors) to clarify whether the absence of HLA-G may “unmask” a favorable effect of CD8^+^ infiltration even in the presence of PD-L1. In this subgroup, tumors with high CD8^+^ TILs density were associated with a significantly prolonged DFS compared with tumors with low CD8^+^ TILs density, whereas this association was not maintained for OS. Taken together, these findings show that when HLA-G is absent, CD8^+^ infiltration may still retain clinical relevance despite PD-L1 expression, at least in terms of relapse risk. The lack of a parallel OS benefit likely reflects the limited sample size of this subgroup, but the overall pattern reinforces the notion that the prognostic and potentially predictive value of CD8^+^ TILs depends on the combined checkpoint landscape rather than on PD-L1 alone. Importantly, PD-L1^+^/HLA-G⁻ tumors with high CD8^+^ density likely represent an “inflamed” microenvironment in which cytotoxic T cells are present but may be functionally restrained, at least in part, by the PD-1/PD-L1 axis. However, the present data are prognostic rather than predictive. Therefore, while this biological context may be compatible with sensitivity to anti–PD-1/PD-L1 therapy, our findings do not demonstrate treatment responsiveness and should not be interpreted as direct evidence of benefit from immune checkpoint blockade. Rather, these results should be considered hypothesis-generating and warrant validation in dedicated predictive studies including patients treated with immunotherapy.

Both PD-L1 and HLA-G are co-up-regulated in a wide spectrum of malignancies, including pancreatic (17), breast (18), renal (19), and colorectal cancer (20). Their expression climbs further when cancer cells are forced to differentiate or exposed to chemotherapeutic stress, thereby reinforcing an immunosuppressive micro-environment and fostering drug resistance (21, 22).

Mechanistically, HLA-G and PD-L1 deploy convergent strategies: both can trigger Fas-dependent apoptosis of effector lymphocytes (23), circulate as immunoregulatory exosomes that blunt T- and NK-cell activity (24, 25), and spread laterally by trogocytosis (26, 27).

Therapeutically, this interplay helps explain why some tumors escape anti-PD-1/PD-L1 therapy: elevated circulating HLA-G and its receptor ILT-2 correlate with resistance (28) and HLA-G-rich PD-1⁻ ILT2^+^ cytotoxic T cells in renal cancer are selectively inhibited unless HLA-G is blocked (29).

Our study provides clinical evidence that HLA-G expression modifies the prognostic impact of CD8^+^ TILs in NSCLC. Assessing HLA-G status alongside PD-L1 and CD8^+^ T-cell density may refine prognostic stratification and identify patients who could benefit from HLA-G-targeted therapies. Notably, PD-L1^-^/HLA-G^+^ tumors could be rational candidates for therapies targeting the HLA-G/ILT2–ILT4 pathway, either alone (to restore CD8^+^ functionality in otherwise PD-L1–negative disease) or in combination strategies aimed at overcoming layered immunosuppression, supporting the broader concept of checkpoint redundancy rather than a single dominant escape mechanism.

Recent pre-clinical evidence supports this notion. Lin et al. created a nanobody-based trispecific T-cell engager that connects CD3-positive T cells to tumor cells expressing PD-L1 and/or HLA-G; this construct markedly enhanced peripheral-blood mononuclear-cell cytotoxicity *in vitro* and, in humanized NSCLC mice, produced deeper tumor regression and longer survival than mono- or bispecific comparators, without additional toxicity (30). Complementing these findings, Chen et al. showed that sub-lethal pemetrexed consistently up-regulates and glycosylates both checkpoints across NSCLC cell lines and that combining anti-PD-L1 and anti-HLA-G antibodies with pemetrexed yields the strongest CTL activation and tumor control *in vitro* and in xenografts (22). Collectively, these studies indicate that dual blockade of PD-L1 and HLA-G, delivered either via multispecific engagers or in conjunction with checkpoint-sensitizing chemotherapy, can overcome redundant immune-evasion mechanisms and may broaden the cohort of patients who achieve durable benefit from immunotherapy. In addition to the preclinical research above, clinical trials in small groups are also in progress (12).

Limitations of our study include its retrospective design and relatively small number of HLA-G–positive cases. Nevertheless, the findings support a model in which tumor-intrinsic HLA-G expression defines an immune-privileged niche that negates the beneficial effect of cytotoxic T-cell infiltration. We also acknowledge the relatively small number of HLA-G^+^ tumors (50/314, 16%). This became particularly relevant in subgroup analyses after stratification by CD8 density and PD-L1 status, as the HLA-G^+^/CD8-low and HLA-G^+^/CD8-high groups comprised only 19 and 31 cases, respectively. Therefore, the lack of significant survival stratification by CD8 within HLA-G^+^ tumors should be interpreted cautiously, since limited statistical power may have contributed to these negative findings.

A further limitation of this study is the use of TMA-based sampling. To partially address spatial heterogeneity, we sampled five 1-mm cores from different tumor areas for each case. This approach was based on our previous NSCLC study on PD-L1 heterogeneity, in which the mean value across cores showed 95.9% concordance with whole sections at a 1% cutoff, supporting the use of multi-core sampling to improve representativeness (31). However, TMA-based assessment cannot fully recapitulate whole-section evaluation, and immune-related biomarkers such as HLA-G, PD-L1, and CD8^+^ infiltrates may still show substantial intratumoral variability. Since we did not perform a formal core-by-core concordance analysis for HLA-G in the present cohort, we cannot exclude that focal or discordant expression across cores may have affected classification in a subset of tumors.

In this work, we used clone 4H84, one of the most widely adopted anti-HLA-G antibodies in the cancer literature, including prior lung cancer studies (11). At the same time, we acknowledge that HLA-G IHC remains methodologically heterogeneous and that antibody specificity has been debated, with reports of potential non-specificity or cross-reactivity of 4H84 under some experimental conditions. In this context, we chose a conservative scoring strategy based exclusively on membranous staining in tumor cells, regardless of intensity, with a 1% cutoff for positivity. Nevertheless, further standardization of HLA-G antibodies and scoring approaches will be important for cross-study comparability.

Finally, we did not evaluate the infiltration and the relative density of CD4^+^ T helper (Th) cells and its relationship with HLA-G expression. Given the role of HLA-G in favoring Th2 over Th1 polarization, it is reasonable to anticipate that HLA-G expression may also modify the tumor microenvironment toward a pro-tumor state by suppressing Th1-mediated anti-tumor responses (32).

In conclusion, our analysis underscores the importance of incorporating HLA-G into the immunophenotypic assessment of NSCLC and further support the rationale for developing therapies targeting HLA-G and its receptors, especially in combination with other immunotherapy strategies.

## Data Availability

Generated dataset cannot be shared due to IRB restrictions. Requests to access the datasets should be directed to enrico_munari@yahoo.it.

## Supplementary material

Supplementary Figure 1 Kaplan-Meier curves for disease-free survival and overall survival for individual parameters: CD8 **(A, B)**, PD-L1 **(C, D)** and HLA-G **(E, F)**.

Supplementary Table 1 Clinicopathological characteristics of patients with non-small cell lung cancer, stratified according to PD-L1 expression.

Supplementary Table 2 Subgroup sizes according to HLA-G expression/CD8 TILs density and event counts.

## References

[1] Morandi F Fainardi E Rizzo R Rouas-Freiss N . The role of HLA-class Ib molecules in immune-related diseases, tumors, and infections. J Immunol Res. (2014) 2014:1–2. doi: 10.1155/2014/231618 24982921 PMC4058464

[2] Kovats S Main E Librach C Stubblebine M Fisher S DeMars R . A class I antigen, HLA-G, expressed in human trophoblasts. Sci (1979). (1990) 248:220–3. doi: 10.1126/SCIENCE.2326636 2326636

[3] Le Bouteiller P Lenfant F . Antigen-presenting function(s) of the non-classical HLA-E, -F and -G class I molecules: The beginning of a story. Res Immunol. (1996) 147:301–13. doi: 10.1016/0923-2494(96)89643-X 8876058

[4] van der Meer A Lukassen H van Cranenbroek B Weiss E Braat D van Lierop M . Soluble HLA-G promotes Th1-type cytokine production by cytokine-activated uterine and peripheral natural killer cells. Mol Hum Reprod. (2007) 13:123–33. doi: 10.1093/MOLEHR/GAL100 17121749

[5] Moretta A Bottino C Vitale M Pende D Cantoni C Mingari M . Activating receptors and coreceptors involved in human natural killer cell-mediated cytolysis. Annu Rev Immunol. (2001) 19:197–223. doi: 10.1146/annurev.immunol.19.1.197 11244035

[6] Kochan G Escors D Breckpot K Guerrero-Setas D . Role of non-classical MHC class I molecules in cancer immunosuppression. Oncoimmunology. (2013) 2:e26491. doi: 10.4161/onci.26491 24482746 PMC3894240

[7] Paul P Rouas-Freiss N Khalil-Daher I Moreau P Riteau B Gal FL . HLA-G expression in melanoma: A way for tumor cells to escape from immunosurveillance. Proc Natl Acad Sci USA. (1998) 95:4510–5. doi: 10.1073/PNAS.95.8.4510 9539768 PMC22520

[8] Yie S Yang H Ye S Li K Dong D Lin X . Expression of human leucocyte antigen G (HLA-G) is associated with prognosis in non-small cell lung cancer. Lung Cancer. (2007) 58:267–74. doi: 10.1016/j.lungcan.2007.06.011 17673327

[9] van de Water R Krijgsman D Houvast R Vahrmeijer A Kuppen P . A critical assessment of the association between HLA-G expression by carcinomas and clinical outcome. Int J Mol Sci. (2021) 22:8265. doi: 10.3390/ijms22158265 34361031 PMC8347921

[10] Dong D Yie S Li K Li F Xu Y Xu G . Importance of HLA-G expression and tumor infiltrating lymphocytes in molecular subtypes of breast cancer. Hum Immunol. (2012) 73:998–1004. doi: 10.1016/j.humimm.2012.07.321 22841927

[11] Bartolome J Molto C Benitez-Fuentes J Fernandez-Hinojal G Manzano A Perez-Segura P . Prognostic value of human leukocyte antigen G expression in solid tumors: a systematic review and meta-analysis. Front Immunol. (2023) 14:1165813. doi: 10.3389/fimmu.2023.1165813 37275862 PMC10232772

[12] Wang S Wang J Xia Y Zhang L Jiang Y Liu M . Harnessing the potential of HLA-G in cancer therapy: advances, challenges, and prospects. J Transl Med. (2024) 22:130. doi: 10.1186/s12967-024-04938-w 38310272 PMC10838004

[13] Munari E Marconi M Querzoli G Lunardi G Bertoglio P Ciompi F . Impact of PD-L1 and PD-1 expression on the prognostic significance of CD8+ tumor-infiltrating lymphocytes in non-small cell lung cancer. Front Immunol. (2021) 12:680973. doi: 10.3389/fimmu.2021.680973 34122444 PMC8187779

[14] Amin M Edge S Greene F Byrd D Brookland R Washington M . AJCC Cancer Staging Manual (8th Edition). Chicago, IL: Springer International Publishing: American Joint Commission on Cancer (2017).

[15] Bankhead P Loughrey M Fernández J Dombrowski Y McArt D Dunne P . QuPath: Open source software for digital pathology image analysis. Sci Rep. (2017) 7:16878. doi: 10.1038/s41598-017-17204-5 29203879 PMC5715110

[16] Yan Q Li S He L Chen N . Prognostic implications of tumor-infiltrating lymphocytes in non-small cell lung cancer: a systematic review and meta-analysis. Front Immunol. (2024) 15:1476365. doi: 10.3389/fimmu.2024.1476365 39372398 PMC11449740

[17] Hiraoka N Ino Y Hori S Yamazaki-Itoh R Naito C Shimasaki M . Expression of classical human leukocyte antigen class I antigens, HLA-E and HLA-G, is adversely prognostic in pancreatic cancer patients. Cancer Sci. (2020) 111:3057–70. doi: 10.1111/CAS.14514 32495519 PMC7419048

[18] Steven A Seliger B . The role of immune escape and immune cell infiltration in breast cancer. Breast Care. (2018) 13:16–21. doi: 10.1159/000486585 29950962 PMC6016054

[19] Rouas-Freiss N LeMaoult J Verine J Tronik-Le Roux D Culine S Hennequin C . Intratumor heterogeneity of immune checkpoints in primary renal cell cancer: Focus on HLA-G/ILT2/ILT4. Oncoimmunology. (2017) 6:e1342023. doi: 10.1080/2162402X.2017.1342023 28932645 PMC5599087

[20] Chen Q-Y Chen Y-X Han Q-Y Zhang J-G Zhou W-J Zhang X . Prognostic significance of immune checkpoints HLA-G/ILT-2/4 and PD-L1 in colorectal cancer. Front Immunol. (2021) 12:679090. doi: 10.3389/fimmu.2021.679090 34054869 PMC8155601

[21] Ullah M Meziani S Shah S Kaci R Pimpie C Pocard M . Differentiation of cancer cells upregulates HLA-G and PD-L1. Oncol Rep. (2020) 43:1797–804. doi: 10.3892/OR.2020.7572 32236615 PMC7160553

[22] Chen M Hung M Pan C Huang S Jan C Li Y . Pemetrexed combined with dual immune checkpoint blockade enhances cytotoxic T lymphocytes against lung cancer. Cancer Sci. (2023) 114:2761–73. doi: 10.1111/CAS.15806 37017116 PMC10323078

[23] Contini P Ghio M Poggi A Filaci G Indiveri F Ferrone S . Soluble HLA-A,-B,-C and -G molecules induce apoptosis in T and NK CD8+ cells and inhibit cytotoxic T cell activity through CD8 ligation. Eur J Immunol. (2003) 33:125–34. doi: 10.1002/IMMU.200390015 12594841

[24] Kim D Kim H Choi Y Kim S Lee J-E Sung K . Exosomal PD-L1 promotes tumor growth through immune escape in non-small cell lung cancer. Exp Mol Med. (2019) 51:1–13. doi: 10.1038/s12276-019-0295-2 31399559 PMC6802663

[25] Riteau B Faure F Menier C Viel S Carosella E Amigorena S . Exosomes bearing HLA-G are released by melanoma cells. Hum Immunol. (2003) 64:1064–72. doi: 10.1016/j.humimm.2003.08.344 14602237

[26] Gary R Voelkl S Palmisano R Ullrich E Bosch J Mackensen A . Antigen-specific transfer of functional programmed death ligand 1 from human APCs onto CD8+ T cells via trogocytosis. J Immunol. (2012) 188:744–52. doi: 10.4049/jimmunol.1101412 22174448

[27] LeMaoult J Caumartin J Daouya M Favier B Le Rond S Gonzalez A . Immune regulation by pretenders: Cell-to-cell transfers of HLA-G make effector T cells act as regulatory cells. Blood. (2007) 109:2040–8. doi: 10.1182/BLOOD-2006-05-024547 17077329

[28] Mandel I Haves Ziv D Goldshtein I Peretz T Alishekevitz D Fridman Dror A . BND-22, a first-in-class humanized ILT2-blocking antibody, promotes antitumor immunity and tumor regression. J Immunother Cancer. (2022) 10:e004859. doi: 10.1136/jitc-2022-004859 36096532 PMC9472153

[29] Dumont C Jacquier A Verine J Noel F Goujon A Wu C-L . CD8+PD-1-ILT2+ T cells are an intratumoral cytotoxic population selectively inhibited by the immune-checkpoint HLA-G. Cancer Immunol Res. (2019) 7:1619–32. doi: 10.1158/2326-6066.CIR-18-0764 31451484

[30] Lin Y-C Chen M-C Huang S-W Chen Y Ho J Lin F . Targeting dual immune checkpoints PD-L1 and HLA-G by trispecific T cell engager for treating heterogeneous lung cancer. Adv Sci (Weinh). (2024) 11:e2309697. doi: 10.1002/advs.202309697 39234811 PMC11538689

[31] Munari E Zamboni G Lunardi G Marchionni L Marconi M Sommaggio M . PD-L1 expression heterogeneity in non–small cell lung cancer: Defining criteria for harmonization between biopsy specimens and whole sections. J Thorac Oncol. (2018) 13:1113–20. doi: 10.1016/j.jtho.2018.04.017 29704674

[32] Martín-Villa J Vaquero-Yuste C Molina-Alejandre M Juarez I Suárez-Trujillo F López-Nares A . HLA-G: Too much or too little? Role in cancer and autoimmune disease. Front Immunol. (2022) 13:796054. doi: 10.3389/fimmu.2022.796054 35154112 PMC8829012

