# Correction: HLA-G expression in non-small cell lung cancer: prognostic significance and interplay with PD-L1 and CD8+ tumor-infiltrating lymphocytes

**DOI:** 10.3389/fimmu.2026.1917753

**Published:** 2026-07-13

**Authors:** Giulia Querzoli, Giuseppe Bogina, Marcella Marconi, Nicola Tumino, Paola Vacca, Luisella Righi, Sara Pilotto, Anna Caliò, Luca Cima, Stefano Gobbo, Matthew J. Cecchini, Gaetano Paolino, Francesco Ciompi, Roberto S. Accolla, Aldo Scarpa, Gianluigi Lunardi, Emanuela Marcenaro, Lorenzo Moretta, Giuseppe Zamboni, Enrico Munari

**Affiliations:** 1 Pathology Unit, IRCCS Azienda Ospedaliero-Universitaria di Bologna, Bologna, Italy; 2 Pathology Unit, IRCCS Sacro Cuore Don Calabria Hospital, Negrar di Valpolicella, VR, Italy; 3 Innate Lymphoid Cells Unit, Immunology Research Area, Bambino Gesù Children’s Hospital, IRCCS, Rome, Italy; 4 Pathology Unit, Department of Oncology, University of Torino at San Luigi Hospital, Orbassano, Italy; 5 Department of Engineering for Innovation Medicine (DIMI), University of Verona and Verona University Hospital Trust, Verona, Italy; 6 Department of Diagnostic and Public Health, Section of Pathology, University of Verona and Verona University Hospital Trust, Verona, Italy; 7 Pathology Unit, Verona University Hospital Trust, Verona, Italy; 8 Department of Pathology and Laboratory Medicine, Schulich School of Medicine and Dentistry, Western University, London, ON, Canada; 9 Pathology Unit, ASST Spedali Civili di Brescia, Brescia, Italy; 10 Department of Pathology, Radboud University Medical Center, Nijmegen, Netherlands; 11 Laboratories of General Pathology and Immunology “Giovanna Tosi”, Department of Medicine and Technological Innovation, University of Insubria, Varese, Italy; 12 Laboratory Medicine, IRCCS Sacro Cuore Don Calabria Hospital, Negrar di Valpolicella, VR, Italy; 13 Department of Experimental Medicine, University of Genova, Genova, Italy; 14 IRCCS Azienda Ospedaliera Metropolitana (AOM), Genova, Italy; 15 Tumor Immunology Unit, Bambino Gesù Children’s Hospital, IRCCS, Rome, Italy

**Keywords:** HLA-G, immune evasion, non-small cell lung cancer, PD-L1, tumor-infiltrating lymphocytes

The funder European Union – Next Generation EU – NRRP M6C2 – Investment 2.1 “Enhancement and strengthening of biomedical research in the NHS” by the Italian Ministry of Health, PNRR-POC-2023-12377131, to PV and EM was erroneously omitted.

The original version of this article has been updated.

