# Supplementary figures and images for: HLA-G expression in non-small cell lung cancer: prognostic significance and interplay with PD-L1 and CD8+ tumor-infiltrating lymphocytes
Source: Front Immunol. 2026 Jun 12;17:1732852. doi: 10.3389/fimmu.2026.1732852 (PMC13303491; doi:10.3389/fimmu.2026.1732852)

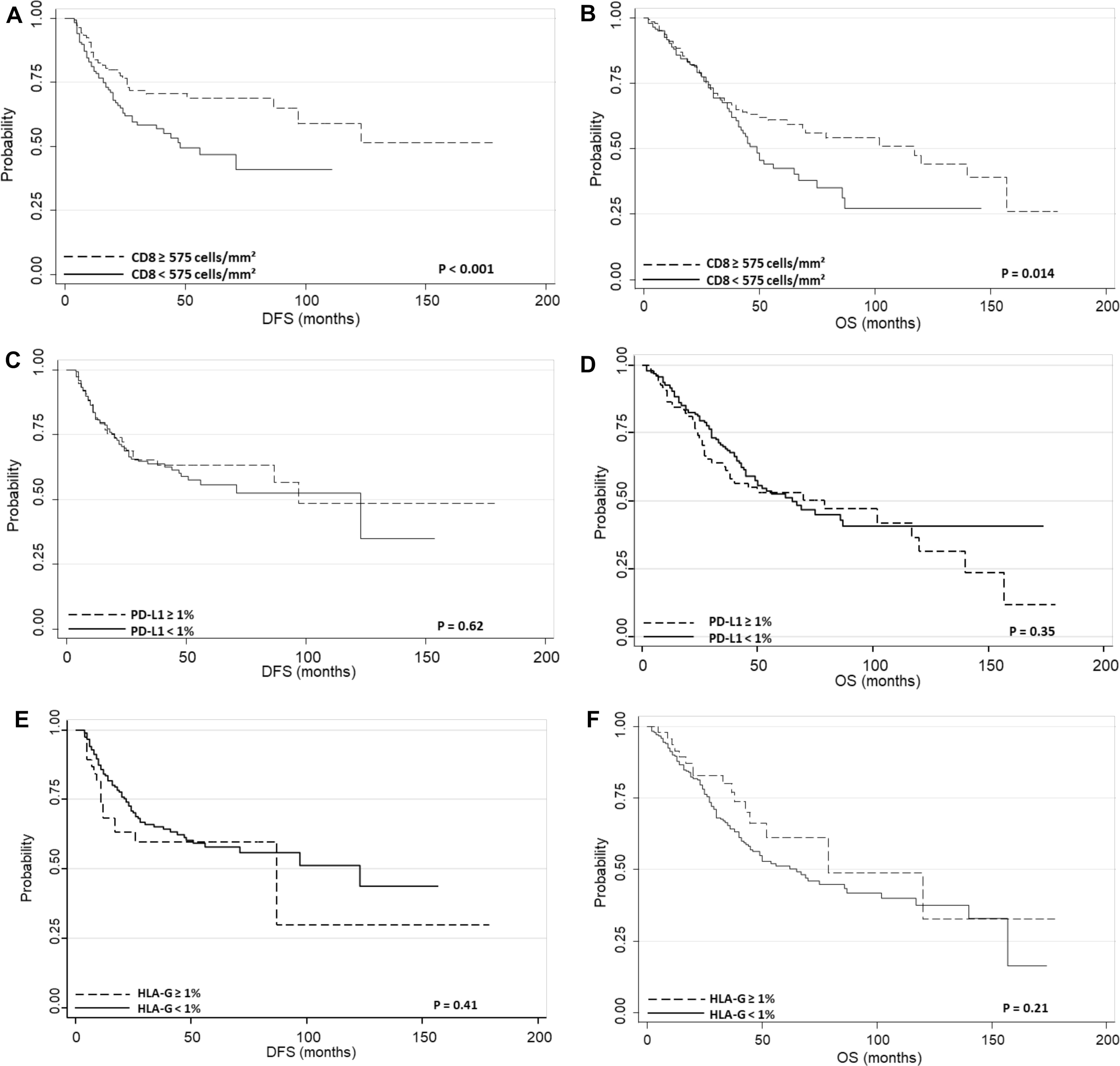

Supplement: Supplementary Figure 1 — Kaplan-Meier curves for disease-free survival and overall survival for individual parameters: CD8 (A, B), PD-L1 (C, D) and HLA-G (E, F). [file Image1.jpeg]
