# Supplementary material for: HLA-G expression in non-small cell lung cancer: prognostic significance and interplay with PD-L1 and CD8+ tumor-infiltrating lymphocytes
Source: Front Immunol. 2026 Jun 12;17:1732852. doi: 10.3389/fimmu.2026.1732852 (PMC13303491; doi:10.3389/fimmu.2026.1732852)
Supplement: Supplementary Table 1 — Clinicopathological characteristics of patients with non-small cell lung cancer, stratified according to PD-L1 expression. [file Table1.docx]

**Supplementary table 1:** Clinicopathological characteristics of patients with non-small cell lung cancer, stratified according to HLA-G expression

| **Variables** | **Overall (%)** | **PD-L1** | |  |
| --- | --- | --- | --- | --- |
|  |  | **< 1% (%)** | **≥ 1% (%)** | **P value** |
| **Patients** | 314 | 208 (66.2) | 106 (33.8) |  |
| **Sex** |  |  |  |  |
| Male | 221 (70.4) | 138 (66.3) | 83 (78.3) | *0.03* |
| Female | 93 (29.6) | 70 (33.7) | 23 (21.7) |  |
| **Age** (years) |  |  |  |  |
| ≤ 70 | 163 (51.9) | 106 (51) | 57 (53.8) | *0.64* |
| > 70 | 151 (48.1) | 102 (49) | 49 (46.2) |  |
| **Histology** |  |  |  |  |
| Adenocarcinoma | 224 (71.4) | 159 (76.4) | 65 (61.3) | *0.009* |
| SCC | 72 (22.9) | 37 (17.8) | 35 (33) |  |
| Others | 18 (5.7) | 12 (5.8) | 6 (5.7) |  |
| **Surgery** |  |  |  |  |
| Wedge/Segmentectomy | 56 (17.8) | 38 (18.3) | 18 (17) | *0.75* |
| Lobectomy | 244 (77.7) | 162 (77.8) | 88 (77.4) |  |
| Pneumonectomy | 14 (4.5) | 8 (3.9) | 6 (5.6) |  |
| **TNM Stage** |  |  |  |  |
| I | 159 (50.6) | 108 (51.9) | 51 (48.1) | *0.83* |
| II | 80 (25.5) | 54 (26) | 26 (24.6) |  |
| III | 53 (16.9) | 33 (15.9) | 20 (18.8) |  |
| IV | 16 (5.1) | 10 (4.8) | 6 (5.7) |  |
| Unknow | 6 (1.9) | 3 (1.4) | 3 (2.8) |  |
| **Adjuvant Treatment** |  |  |  |  |
| No | 242 (77.1) | 163 (78.4) | 79 (74.5) | *0.33* |
| Yes | 44 (14) | 25 (12) | 19 (17.9) |  |
| Unknow | 28 (8.9) | 20 (9.6) | 8 (7.6) |  |
|  |  |  |  |  |
| **CD8** |  |  |  |  |
| < 575 cells/mm² | 154 (49) | 122 (58.7) | 32 (30.3) | *<0.001* |
| ≥ 575 cells/mm² | 160 (51) | 86 (41.3) | 74 (69.7) |  |
