# Supplementary material for: HLA-G expression in non-small cell lung cancer: prognostic significance and interplay with PD-L1 and CD8+ tumor-infiltrating lymphocytes
Source: Front Immunol. 2026 Jun 12;17:1732852. doi: 10.3389/fimmu.2026.1732852 (PMC13303491; doi:10.3389/fimmu.2026.1732852)
Supplement: Supplementary Table 2 — Subgroup sizes according to HLA-G expression/CD8 TILs density and event counts. [file Table2.docx]

**Supplementary table 2:** subgroup sizes according to HLA-G expression / CD8 TILs density and event counts

|  | **recurrence** | | |  |
| --- | --- | --- | --- | --- |
| **HLA-G/CD8 status** | Yes (%) | No (%) | . | Total |
| HLA-G^-^/CD8low | 54 (40) | 52 (38.5) | 29 (21.5) | 135 |
| HLA-G^-^/CD8high | 63 (48.8) | 25 (19.4) | 41 (31.8) | 129 |
| HLA-G^+^/CD8low | 11 (57.9) | 6 (31.6) | 2 (10.5) | 19 |
| HLA-G^+^/CD8high | 11 (35.5) | 11 (35.5) | 9 (29) | 31 |
| Total | 139 (44.3) | 94 (29.9) | 81 (25.8) | 314 |

|  | **death** | | |  |
| --- | --- | --- | --- | --- |
| **HLA-G/CD8 status** | Yes (%) | No (%) | . | Total |
| HLA-G^-^/CD8low | 67 (49.6) | 60 (44.4) | 8 (6) | 135 |
| HLA-G^-^/CD8high | 75 (58) | 44 (34) | 10 (8) | 129 |
| HLA-G^+^/CD8low | 13 (68.4) | 6 (31.6) | 0 | 19 |
| HLA-G^+^/CD8high | 18 (58) | 10 (32.3) | 3 (9.7) | 31 |
| Total | 173 (55.1) | 120 (38.2) | 21 (6.7) | 314 |
